# Proteome and Phosphoproteome Analyses Reveal the Kinase Regulatory Network Involved in Glycogen Synthesis Kinase 3β

**DOI:** 10.3389/fgene.2021.657140

**Published:** 2021-04-07

**Authors:** Mingyang Hu, Jiuyuan Fang, Huijuan Wang, Sijie Zhou

**Affiliations:** ^1^The First Affiliated Hospital of Zhengzhou University, Zhengzhou, China; ^2^Zhengzhou University School of Medical Sciences, Zhengzhou, China; ^3^School of Basic Medical Sciences, Zhengzhou University, Zhengzhou, China

**Keywords:** GSK-3β, phosphoproteome, proteome, podocytes, diabetic nephropathy

## Abstract

Diabetic nephropathy is the most common chronic kidney disease in the world and the main cause of end-stage renal disease (ESRD). The structural integrity of podocytes is fundamental to the normal function of the glomerulus, and the role of glycogen synthase kinase 3β (GSK-3β) in podocytes is complicated. A thorough understanding of GSK-3β is crucial to understand the mechanism of diabetic nephropathy. To analyze the roles of GSK-3β in podocytes, GSK-3β knockdown lentivirus by clustered regularly interspaced short palindromic repeats (CRISPR)–CRISPR-associated protein (Cas)9 was applied to establish stable cell lines. Mass spectrometry was utilized to search for differentially expressed proteins. Consequently, we found 34 proteins with higher levels and 115 proteins with lower levels in GSk-3β knockdown cells than in control cells and identified 581 phosphosites with higher phosphorylation levels and 288 phosphosites with lower phosphorylation levels. We performed functional enrichment analysis of these proteins and phosphorylated proteins based on public databases. Enrichment analysis revealed that GSK-3β participates in the spliceosome, Hippo signaling pathway, actin binding, structural molecule activity, and other pathways. Then, we used motif analysis of phosphate sites to determine 89 conserved motifs based on 1,068 phosphoserine (pS) sites and 15 conserved motifs in view of 104 phosphothreonine (pT) sites. Additionally, protein–protein interaction network analysis was carried out using the STRING database. Cytoscape’s add-on Molecular Complex Detection (MCODE) was used to analyze key and core protein groups. In quantitative differential protein analysis, four MCODEs were obtained, and 22 MCODEs were obtained in the analysis of the phosphoproteome of differentially expressed proteins. Finally, we analyzed the kinase regulatory network in podocytes after GSK-3β knockdown and identified 299 protein kinases and 3,460 significantly changed phosphorylation modification sites on 1,574 proteins. These results will be valuable for further research on GSK-3β.

## Introduction

Diabetic nephropathy is the most common chronic kidney disease and the leading cause of end-stage renal disease (ESRD) worldwide. In the past two decades, diabetic nephropathy (DN) has become the main cause of chronic kidney disease (CKD) and renal failure, and its morbidity and mortality have also increased enormously (Heerspink et al., 2019). DN is a microvascular complication of type 1 and type 2 diabetes (T1D and T2D), which is characterized by dysfunction and glomerular filtration barrier damage, leading to albuminuria. The structural integrity of podocytes is vital to the normal function of the glomerulus, and podocyte damage is the basis for the progression of several kidney diseases (Pavenstädt et al., 2003). Much of the research in podocytes in recent years has examined whether enhanced glycogen synthase kinase 3β (GSK-3β) activity mediates podocyte apoptosis (Paeng et al., 2014) and autonomous injury (Li et al., 2016) under diabetic conditions in proteinuria glomerulopathy.

As a serine/threonine kinase, GSK-3β plays an indispensable role in adjusting sugar metabolism, cellular inflammatory response, nerve and cardiac function, and reproductive function (Jope et al., 2007; Wang et al., 2015; Zhou et al., 2016, 2018). It is functional in the regulation of multiple cell pathways, including cell proliferation (Shen et al., 2015), differentiation (Yang et al., 2018), and apoptosis (Peixoto et al., 2015). The expression and activity of GSK-3β in the renal tissue of patients with diabetic nephropathy have been reported to be significantly regulated (Guo et al., 2014). GSK-3β is a fundamental regulator of many signaling pathways (Doble and Woodgett, 2003). The expression of GSK-3β with low levels can reduce the expression of β-catenin and Snail (Guo et al., 2014) and reverse the EMT of podocytes induced by high glucose (HG).

It is now well established from a variety of studies that GSK-3β in diabetic nephropathy mainly converge Wnt/β-catenin, PI3K/Akt/GSK-3β, epithelial–mesenchymal transition, etc (Cheng et al., 2016). The role of GSK-3β in podocytes is multifaceted. An in-depth study of GSK-3β helps to better understand the mechanism of diabetic nephropathy and provides new insights for the prevention and treatment of the disease. There are no related studies on GSK-3β, which could function as a kinase to manipulate the downstream protein regulatory network in podocytes, so we used phosphorylated proteomics to discuss it. Protein phosphorylation, as one of the most characterized posttranscriptional modifications, is a rapid and reversible mechanism that may participate in all cellular processes and in regulating various metabolic processes (Hunter, 1995). It is involved in signal transduction and indirect regulation of metabolism and directly affects metabolic enzymes by changing protein conformation (Humphrey et al., 2015).

This article explores the changes in protein and phosphorylated protein in podocytes after GSK-3β knockdown to learn the influence affected by GSK-3β and probes new ways of preventing or treating diabetic nephropathy. A total of 581 proteins with higher levels and 288 proteins with lower levels in the lentivirus-mediated GSK-3β knockdown group than in the control group were identified. Regarding differentially expressed proteins of the quantitative proteome [quantitative differentially expressed proteins (QDEPs)], the Kyoto Protocol Encyclopedia of Genes and Genomes (KEGG) database was enriched in axon guidance and actin cytoskeleton regulation. The Hippo signaling pathway, spliceosome, and other pathways were enriched in the phosphorylated proteome. Moreover, 104 motifs were enriched in view of the software MoMo (Cheng et al., 2019). Furthermore, 156,515 regulatory relationships between 299 protein kinases and 3,460 significantly changed phosphorylation modification sites on 1,574 proteins were identified. To the best of our knowledge, this is the first study to conduct a global analysis of GSK-3β-dependent phosphorylation in podocytes.

## Materials and Methods

### Cell Culture

Mouse podocyte clone 5 (MPC5) used in the cell experiment was purchased from Shanghai Kwaisai Biotechnology Co., Ltd. Cells were cultured in T25 cell culture flasks with medium consisting of 1% penicillin, 10% fetal bovine serum, and 90% low-glycemic DMEM (Shanghai Xiaopeng Biological Technology Co., Ltd). All cells were cultured at 37°C, 5% CO_2_.

### Lentiviral Transfection and Stable Transformation Construction

Glycogen synthase kinase 3β knockdown (CRISPR Cas9 technology) with flag tag and lentiviral vector with flag were purchased from Shanghai Jikai Gene Chemical Technology Co., Ltd. In addition, all viral vectors are resistant to puromycin. The multiplicity of infection (MOI) and the optimal infection conditions of the lentivirus for MPC5 infection were determined by experiments, and finally, an MOI of 15 was selected on the basis of the company’s instructions. First, we spread two six-well plates with 100,000 cells per well. Next, we added knockout GSK-3β lentivirus and control lentivirus in line with the viral vector solution and the MOI value obtained in the preliminary experiment. In addition, we carried out preliminary experiments to determine the best working concentration according to the instructions of puromycin (Dalian Meilun Biotechnology Co., Ltd). Twenty-one concentration gradients were set from 0 to 10 μg/ml, and after 48 h of observation, it was confirmed that 99% of cells without puromycin resistance could be killed at a concentration of 2 μg/ml. After the virus was transfected into the cells for 48 h, diluted puromycin was added to the six-well plate at a concentration of 2 μg/ml. Finally, we removed the culture solution with puromycin and added a culture medium to expand the cells after 48 h of puromycin treatment.

### Western Blotting Analysis

Total protein was extracted from transfected cells using radioimmunoprecipitation assay (RIPA) lysis buffer (Beijing Soleibao Technology Co., Ltd). Protein concentration was quantified using a BCA kit (Shanghai Biyuntian Biotechnology Co., Ltd). All of the cell lysates were denatured by boiling in loading buffer. Ten percent separating gel and 6% concentrated gel were configured according to the instructions of the gel making kit (Shanghai Biyuntian Biotechnology Co., Ltd). Then, we added 20 to 30 μg of protein per well to the channel. The concentrated gel was electrophoresed at 80 V, and the separation gel was electrophoresed at 120 V constant pressure electrophoresis. Then, proteins were separated by sodium dodecyl sulfate-polyacrylamide gel electrophoresis (SDS-PAGE) and transferred to a polyvinylidene fluoride (PVDF) membrane at a voltage of 250 mA for 90 min. Blocking was completed using 5% skim milk/TBST. The film was washed with TBST three times for 5 min, and the primary antibody was added at a ratio of 1:1,000 (the antibody diluent was purchased from Shanghai Biyuntian Biotechnology Co., Ltd) and incubated overnight at 4°C. The next day, after washing the membrane three times with TBST, the membrane was incubated with the secondary antibody at room temperature for 2 h. Finally, protein bands were visualized with electrochemiluminescence (ECL) (Dalian Meilun Biotechnology Co., Ltd) on the AI600 machine (Thermo Fisher Scientific).

### Sample Preparation and Protein Extraction

The validated successfully transfected cells were expanded and cultured at 37°C and 5% CO_2_. We washed the cells with precooled PBS solution several times before preparing the samples, collected the cells with a cell scraper, and placed them in an EP tube. Samples were taken at -80°C. Then, four times the volume of lysis buffer (8 M urea, 1% protease inhibitor, 1% phosphatase inhibitor) was added and analyzed by ultrasonic testing. We centrifuged the sample at 12,000 × *g* for 10 min at 4°C to remove cell debris, transferred the supernatant to a new centrifuge tube, and determined the protein concentration using the BCA kit.

### Trypsin Digestion

For digestion, the protein solution was reduced with 5 mM dithiothreitol for 30 min at 56°C and alkylated with 11 mM iodoacetamide for 15 min at room temperature in darkness. The protein samples were then diluted by adding 100 mM TEAB to a level of urea below 2 M. Finally, trypsin was added at a trypsin–protein mass ratio of 1:50 for the first overnight digestion and a trypsin–protein mass ratio of 1:100 for a second 4-h digestion.

### Tandem Mass Tag Labeling

The peptides digested by trypsin were desalted with Strata X C18 (Phenomenex) and then freeze-dried *in vacuo*. The peptide was dissolved with 0.5 M TEAB and labeled according to the tandem mass tag (TMT) kit operating instructions. Then, one unit of TMT/iTRAQ reagent was thawed and reconstituted in acetonitrile. The peptide mixtures were then incubated for 2 h at room temperature and pooled, desalted, and dried by vacuum centrifugation.

### HPLC Fractionation

The peptides were fractionated by high pH reversed-phase high-performance liquid chromatography (HPLC) using a Thermo Betasil C18 column (5 μm particles, 10 mm ID, 250 mm length). In light of the following operation, the peptide segment has a step gradient of 8 to 32% acetonitrile, pH 9.0, 60 min time separation, and 60 components. Then, the peptides were combined into six fractions and dried by vacuum centrifugation.

### LC-MS/MS Analysis

The peptides were dissolved in mobile phase A of liquid chromatography containing 0.1% formic acid and 2% acetonitrile and then separated using the EASY-nLC 1000 ultrahigh-performance liquid system. Liquid phase B was composed of 0.1% formic acid and 90% acetonitrile. The liquid gradient settings were as follows: 0–20 min, 8∼22% B; 20–33 min, 22∼35% B; 33–37 min, 35∼80% B; and 37–40 min, 80% B. The flow rate was maintained at 550.00 nl/min. The peptides were separated by an ultrahigh-performance liquid system and then injected into the NSI ion source for ionization and then analyzed by QE plus mass spectrometry. The ion source voltage was set to 2.2 kV, and the peptide precursor ions and their secondary fragments were detected and analyzed by high-resolution Orbitrap. The scanning range of the primary mass spectrum was set to 400–1,500 *m*/*z*, and the scanning resolution was set to 70,000.00; the scanning range of the secondary mass spectrum was set to a fixed starting point of 100 *m*/*z*, and the secondary scanning resolution was set to 17,500.00. The data acquisition mode used the data-dependent scanning (DDA) program; that is, the first 20.00 peptide precursor ions with the highest signal intensity were selected after the first scan to enter the HCD collision cell, and 30% of the fragmentation energy was used for fragmentation. Next, to improve the effective utilization of mass spectrometry, the automatic gain control (AGC) was set to 5E4, the signal threshold was set to 6.3E4 ions/s, the maximum injection time was set to 50 ms, and the dynamic rejection time of the tandem mass spectrometry scan was set to 30 s to avoid precursor ion repeated scans.

### Database Search

The MS/MS data obtained were processed through the MaxQuant search engine (v.1.5.2.8). The search parameters were set as follows. (1) The database was Mus_musculus_10090 (17032 sequences), an anti-database was added to calculate the false positive rate (FDR) caused by random matching, and a common contamination library was added to the database to eliminate the contaminating protein from the impact identification results. (2) The restriction enzyme digestion method was set to trypsin/P. (3) The number of missed cleavage sites was set to 2. (4) The minimum peptide length of seven amino acid residues was applied. (5) The maximum modification number of peptides was set to 5. (6) The mass error tolerance was set to 20.0 and 5 ppm. (7) The mass error tolerance of the second fragment ion was 20.0 ppm. (8) Cysteine alkylation carbamidomethyl (C) was set as a fixed modification, and variable modifications were set as [“acetyl (protein N-term),” “oxidation (M),” “deamidation (NQ)”]. (9) The quantitative method was set to TMT-6plex, and the FDR for protein identification and PSM identification was tuned to 1%. The data presented in the study are deposited in the ProteomeXchange Consortium via the PRIDE partner repository, accession number (PXD024543).

### Bioinformatics Methods

We analyzed the differentially expressed proteins from the aspects of Gene Ontology (GO) enrichment, the KEGG enrichment, and domain enrichment. The first was the GO annotation of the protein, which is divided into three categories: biological process, cell composition, and molecular function. Gene Ontology annotations on proteomics were downloaded from the UniProt-GOA database^1^.

The Kyoto Encyclopedia of Genes and Genomes database was used for pathway enrichment analysis. In the KEGG enrichment analysis, we first used the KEGG online service tool KAAS to annotate the submitted protein, and then, we used the KEGG mapper to match the annotated protein into the corresponding pathway in the database.

In addition, the InterPro (providing resources for functional analysis of protein sequence family classification, prediction of structural domains and special sites) database was used to analyze the enrichment of functional domains of differentially expressed proteins. The above analysis used Fischer’s precise double-ended test method to test differentially expressed proteins in the background of the identified proteins. If the *p*-value of the domain unit enrichment test was less than 0.05, the value was considered significant.

Afterward, the differential proteins obtained by phosphorylated proteomics were used to perform motif prediction analysis. This analysis is based on MoMo software, and the motif-x algorithm was used to analyze the motif characteristics of the modified sites. Among them, all the identified phosphorylation modification sites were composed of six amino acids upstream and downstream as the analysis object; the analysis and comparison background were the peptide sequences composed of six amino acids upstream and downstream of all potential phosphorylation modification sites in the species. When the number of peptides in the form of a characteristic sequence is greater than 20 and the statistical test *p*-value is less than 0.000001, the characteristic sequence form is considered to be a motif of modified peptides.

Next, we utilized the STRING database^2^ to visualize the visual network between different proteins and then used the MCODE package^3^ with default parameters in Cytoscape to identify highly interconnected clusters in a network.

Furthermore, a group-based prediction system (GPS5.0)^4^ was adopted to predict the phosphokinase upstream of the phosphorylation site to predict the kinase family that may regulate the specific phosphorylation site. Then, the corresponding kinase protein in the kinase family was obtained by comparison with the kinase sequence in integrated annotations for eukaryotic protein kinases, protein phosphatases, and phosphoprotein-binding domains, ver2.0 (iEKPD2.0), and finally, the protein–protein interaction information (PPI) was used to filter out potential false positive interactions. In addition, we used the iKAP (Mischnik et al., 2016) algorithm to predict kinase activity based on the expression of phosphorylation sites.

## Results

### Global Proteome and Phosphoproteome in MPC5

**FIGURE 1:**
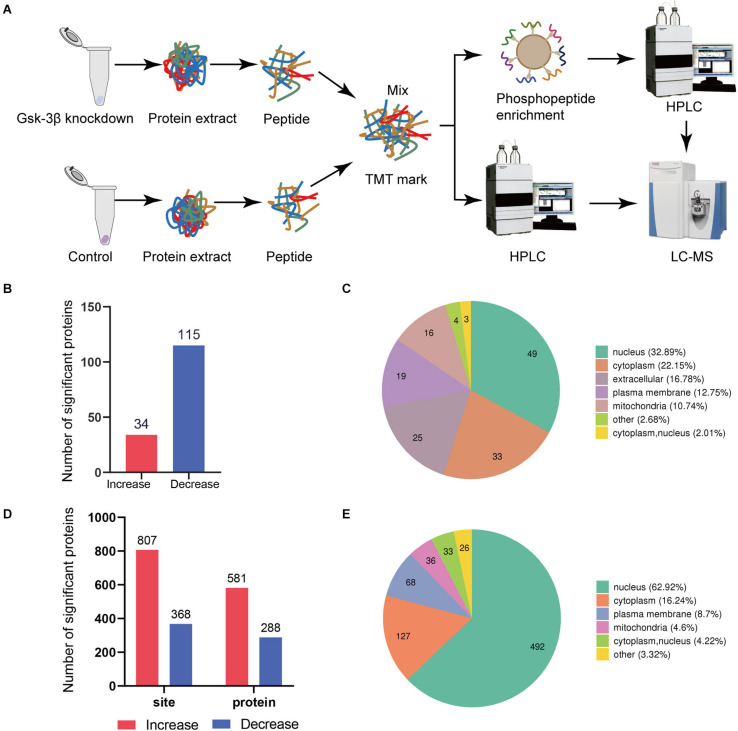
Global proteome and phosphoproteome using LC-MS. **(A)** Schematic diagram of the process of GSK3β knockdown and control group in proteome and phosphorylated proteome. **(B)** Data distribution histogram of QDEPs. Blue indicates decreased differential expression proteins. Red represents increased proteins. **(C)** Quantitative protein subcellular structure localization of differential proteins. The number in the pie chart represents the number of differential proteins in the cell structure. **(D)** Histogram of PDEPs. **(E)** Differential protein subcellular structure localization of the phosphorylated proteome.

The schematic flow is shown in Figure 1A. We applied TMT techniques for labeling, high pH reverse HPLC for fractionation, and TiO_2_ chromatography for phosphopeptide enrichment and utilized liquid chromatography tandem mass spectrometry analysis (Figure 1A). To ensure that the mass spectrometry data is credible, the protein expression levels of GSK-3β, p-GSK-3β, and GSK-3α in GSk-3β knockdown cells than in control cells were determined by Western blot (Supplementary Figure 1). To verify the repeatability of the sample, we performed principal component analysis (PCA) (Supplementary Figure 2).

We recognized that the ratio between the two groups was greater than 1.3 times that of the differentially expressed proteins, so 149 proteins were identified as differentially expressed proteins. A total of 34 proteins with higher levels and 115 proteins with lower levels were found in the lentivirus-mediated GSK-3β knockdown group than in the control group (Figure 1B). A total of 581 phosphosites with higher phosphorylation levels and 288 phosphosites with lower phosphorylation levels were identified in the lentivirus-mediated GSK-3β knockdown group than in the control group (Figure 1D). The analysis of QDEPs and the differentially expressed proteins of the phosphorylated proteome (PDEPs) is helpful to identify proteins that are more closely related to GSK-3β.

In this study, we found that the three proteins (Supplementary Figure 3) Trhde (GSK3B_KD/control ratio = 2.454), Spp1 (GSK3B_KD/control ratio = 1.311), and Mical3 (GSK3B_KD/control ratio = 1.314) had reduced phosphorylation levels but increased total levels. In addition, quantitative proteome and phosphoproteome of differentially expressed protein subcellular location maps (Figures 1C,E) showed that the protein is mostly distributed in the nucleus and cytoplasm. Differential protein localization shows that GSK-3β also plays a role in cell structures such as the extracellular space, plasma membrane, and mitochondria.

### Proteins With a Broad Range of Functions Are Discovered in a GSK-3β-Dependent Manner in Podocytes

**FIGURE 2:**
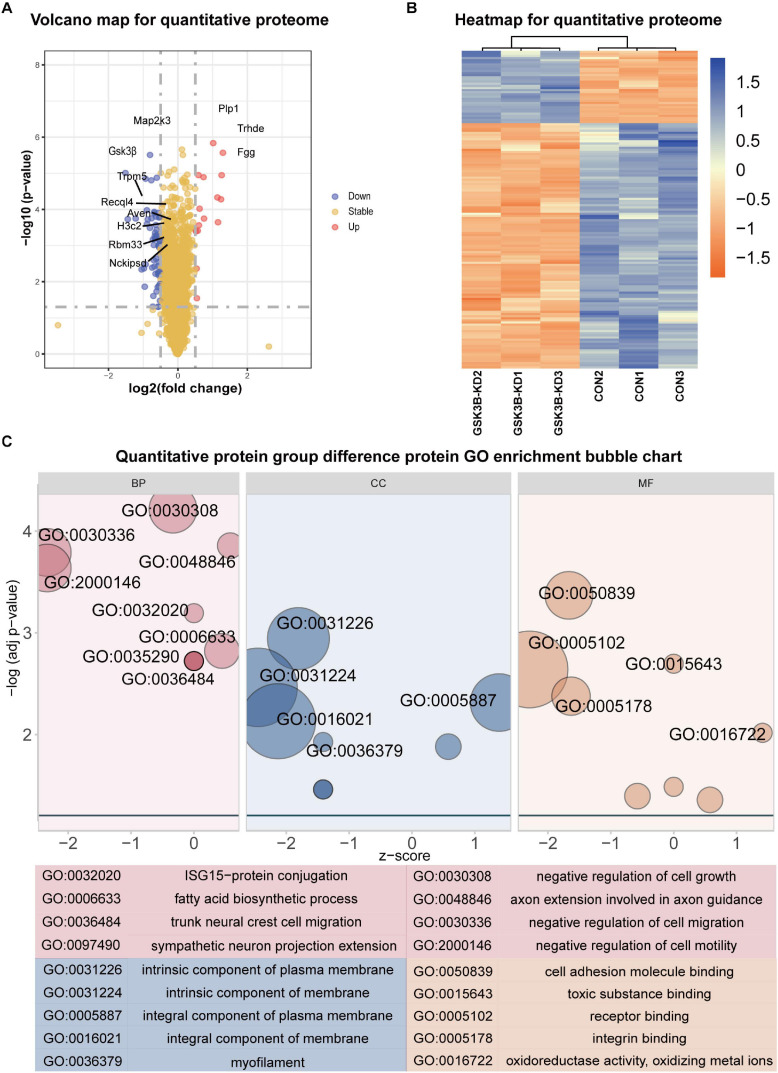
Differential protein analysis and GO enrichment analysis of the quantitative proteome. **(A)** Volcano plot of protein abundance changes in response to GSKSβ knockdown. By *t*-test (–log 10 transformation), the average protein expression ratio (fold change) of 3 replicates (log 2 transformation) between GSK3β and control cells was plotted against the *p*-value. Take Fold change = 1.3 and *P*-value = 0.05 as critical values. **(B)** Heatmap of Quantitative Proteome Differentially Expressed Proteins. **(C)** Gene Ontology (GO) Enrichment for Quantitative Proteome Differentially Expressed Proteins. *Z*-score = (up–down)/vcount.

A total of 5,661 quantitative proteins were identified in the proteome analysis (Supplementary Figure 4A). We defined proteins that were significantly different (Student’s *t*-test, *p* < 0.05) and used the criterion of 1.3-fold or greater change as the criteria to screen candidate proteins and identified 34 proteins with higher levels and 115 proteins with lower levels in the lentivirus-mediated GSK-3β knockdown group than in the control group (Figure 2A). Heatmaps were applied to indicate the expression levels of the differentially expressed proteins screened by the volcano map in three replicate samples of the experimental group and the control group (Figure 2B).

The GO enrichment of differentially expressed proteins obtained is shown in Figure 2C. GO enrichment is mainly divided into three parts: Biological process, cell composition, and molecular function. From the perspective of biological process, differentially expressed proteins are mainly enriched in negative regulation of cell growth, negative regulation of cell migration, axon extension involved in axon guidance, negative regulation of cell motility, ISG15–protein conjugation, etc. From the perspective of cell composition, differentially expressed proteins were mainly enriched in intrinsic components of the plasma membrane, intrinsic components of the membrane, integral components of the plasma membrane, integral components of the membrane, and myofilaments. In the molecular function classification, cell adhesion molecule binding, toxic substance binding, receptor binding, integrin binding, oxidoreductase activity, and oxidizing metal ions were significantly enriched.

### Domain Enrichment, KEGG Enrichment, PPI Network, and Module Analysis of Quantitative Differentially Expressed Proteins

**FIGURE 3:**
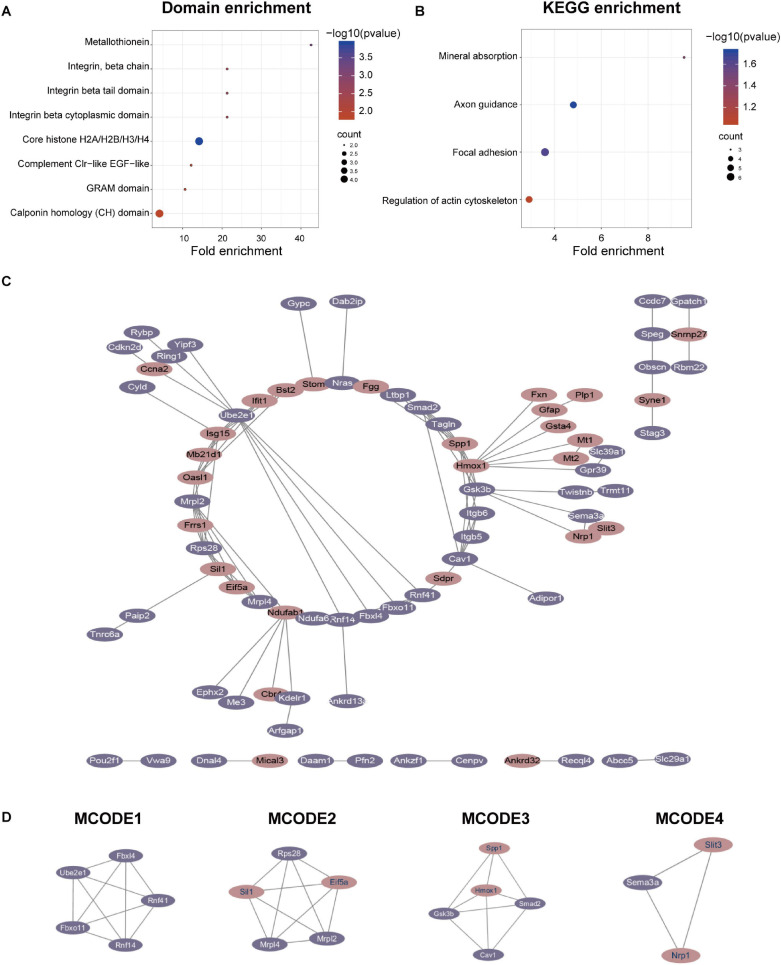
Protein-protein interaction (PPI) network analyses of QDEPs were performed, and the four most significant modules were identified by the molecular complex detection (MCODE) algorithm. **(A)** Domain enrichment of differentially expressed proteins. We used fold enrichment as the abscissa and -log10 (*P*-value) and counted as the ordinate to analyze the domain enrichment of differentially expressed proteins. **(B)** KEGG enrichment of differentially expressed proteins. We used fold enrichment as the abscissa and -log10 (*P*-value) and counted as the ordinate to perform KEGG enrichment analysis on differentially expressed proteins. **(C)** PPI network of differentially expressed proteins. We used STRING and Cytoscape to perform protein-protein interaction analysis and visualize differentially expressed proteins detected by mass spectrometry. **(D)** Four most significant modules of QDEPs. The four most significant modules were yielded by the molecular complex detection (MCODE) algorithm.

We performed domain enrichment and KEGG enrichment on the differentially expressed proteins to obtain a deeper understanding of the role and function of GSK-3β in podocytes. For domain enrichment of differentially expressed proteins (Figure 3A), we identified eight domains: core histone H2A/H2B/H3/H4, metallothionein, integrin beta cytoplasmic domain, integrin beta tail domain, integrin, beta chain, complement Clr-like EGF-like, GRAM domain, and calponin homology (CH) domain. Next, we performed KEGG enrichment for differentially expressed proteins and identified four pathways (Figure 3B) including axon guidance, focal adhesion, mineral absorption, and regulation of actin cytoskeleton. In addition, QDEPs were analyzed for GO, KEGG, and domain enrichment based on the difference expression multiplied QDEPs [Q1 (<0.667), Q2 (0.667–0.769), Q3 (1.3–1.5), Q4 (>1.5)] (Supplementary Figure 5).

Finally, we used STRING and Cytoscape to visualize the interaction between differentially expressed proteins (Figure 3C) and used the MCODE plug-in in Cytoscape software to further analyze the PPI network to obtain four more critical and core protein groups that were more closely connected in the network (Figure 3D). MCODE 1 (MCODE score = 5) consisted 5 nodes and 10 edges, MCODE 2 (MCODE score = 5) consisted 5 nodes and 10 edges, MCODE 3 (MCODE score = 4.5) comprised 5 nodes and 9 edges, and MCODE 4 (MCODE score = 3) comprised 3 nodes and 3 edges. We chose the top 20 hub proteins in the PPI network based on betweenness (Supplementary Table 1), which usually plays a paramount role in network stability due to its high degree of connection/interaction.

### Proteins With a Broad Range of Functions Are Phosphorylated in a GSK-3β-Dependent Manner in Podocytes

**FIGURE 4:**
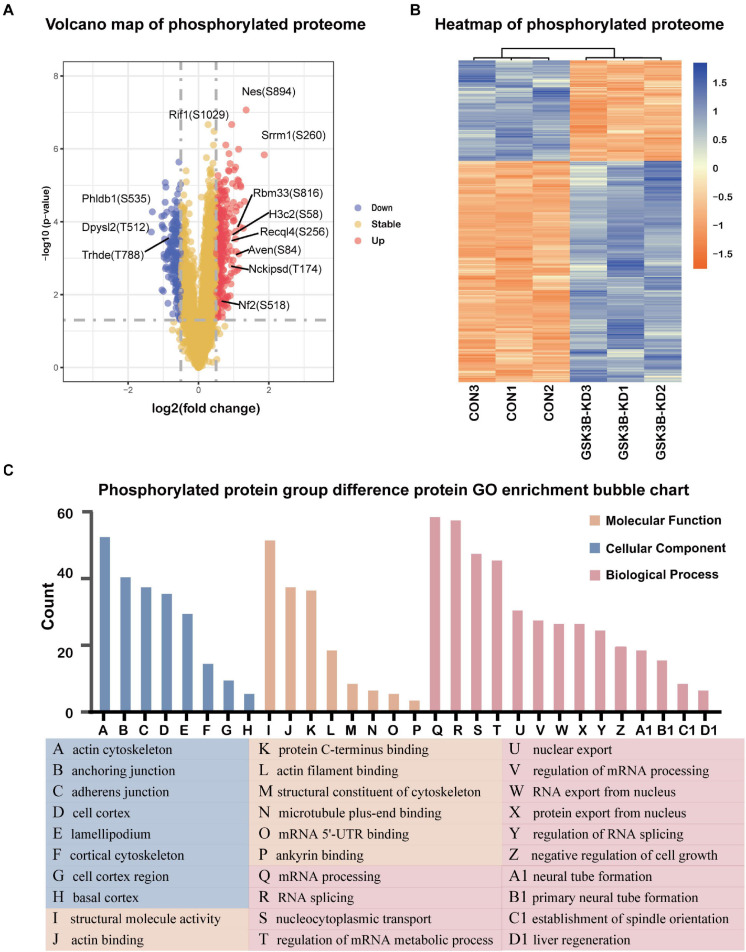
Differentially expressed protein analysis and GO enrichment analysis of the phosphorylated proteome. **(A)** Volcano plot of phosphorylated proteome differentially expressed protein abundance changes in response to GSKSβ knockdown. By *t*-test (–log10 transformation), the average protein expression ratio (fold change) of 3 replicates (log 2 transformation) between GSK3β and control cells was plotted against the *p*-value. A fold change of 1.3 and a *P*-value of 0.05 were considered critical values. **(B)** Heatmap of PDEPs. **(C)** Gene Ontology (GO) Enrichment for PDEPs.

A total of 2,094 quantitative proteins were identified in the experimental group and the control group (Supplementary Figure 4B). Similarly, we identified 581 proteins with higher phosphorylation levels and 288 proteins with lower phosphorylation levels in the lentivirus-mediated GSK-3β knockdown group than in the control group (Figure 4A). Besides, a total of 1,175 phosphosites were identified, of which phosphosites on the serine, threonine, and tyrosine accounted for 90.89% (1,068/1,175), 8.85% (104/1,175), and 0.26% (3/1,175), and we distributed phosphoproteins based on the number of phosphorylation sites per protein (Supplementary Figure 6). The phosphorylation site of Dpysl2/CRMP2 that become less phosphorylated was the most obvious. Research has shown that it plays a role in repairing the integrity of microtubules (Xu et al., 2015). The R package “pheatmap” was used to draw a heatmap (Figure 4B), which shows the expression levels of the differentially expressed proteins at the phosphorylation site screened by the volcano map. Afterward, we performed GO enrichment for phosphorylated differentially expressed proteins (Figure 4C). From the dimension of cell composition, PDEPs are mainly enriched in actin cytoskeleton, cell cortex region, anchoring junction, basal cortex, adherens junction, lamellipodium, cell cortex, and cortical cytoskeleton. Molecular functions were enriched in actin binding, structural molecule activity, microtubule plus-end binding, protein C-terminus binding, mRNA 5′-UTR binding, actin filament binding, structural constituent of cytoskeleton, and ankyrin binding. In the biological process classification, mRNA processing, RNA splicing, nucleocytoplasmic transport, regulation of mRNA metabolic process, nuclear export, regulation of mRNA processing, protein export from nucleus, RNA export from nucleus, and regulation of RNA splicing were significantly enriched.

### Domain Enrichment, KEGG Enrichment, PPI Network, and Module Analysis of Phosphorylated Differentially Expressed Proteins

**FIGURE 5:**
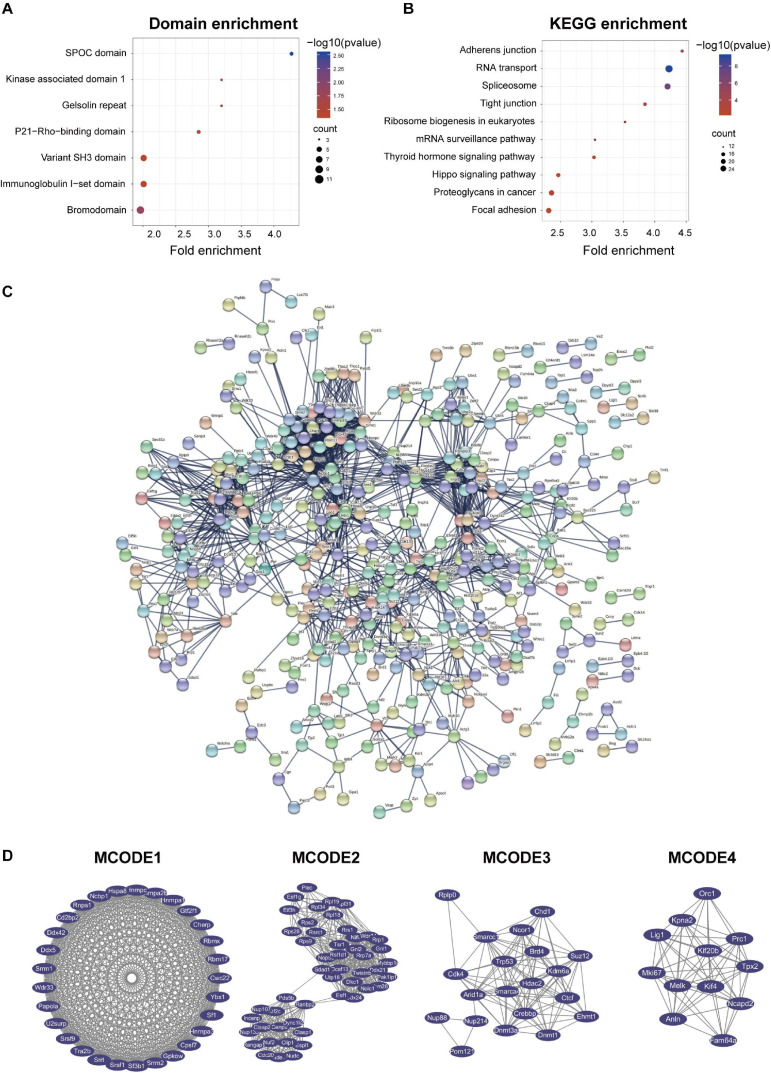
Protein-protein interaction (PPI) network analyses of PDEPs were performed, and the four most significant modules were identified by the molecular complex detection (MCODE) algorithm. **(A)** Domain enrichment of PDEPs. We used fold enrichment as the abscissa, –log10 (*P*-value), and counted as the ordinate for domain enrichment of PDEPs. **(B)** KEGG enrichment of PDEPs. We used fold enrichment as the abscissa and –log10 (*P*-value) as the ordinate to perform KEGG enrichment analysis for PDEPs. **(C)** PPI network of PDEPS. We used STRING and Cytoscape to analyze the protein-protein interactions of the phosphorylated differentially expressed proteins detected by mass spectrometry. **(D)** Four most significant modules of PDEPs. The four most significant modules were yielded by the molecular complex detection (MCODE) algorithm.

Domain enrichment analysis (Figure 5A) and KEGG enrichment analysis were performed to obtain a more comprehensive understanding of GSK-3β in podocytes (Figure 5B) on phosphorylated differentially expressed proteins. Seven domains were enriched in the domain enrichment analysis, namely, SPOC domain, bromodomain, P21-Rho-binding domain, immunoglobulin I-set domain, variant SH3 domain, gelsolin repeat, and kinase-associated domain 1. Subsequently, 10 pathways were enriched, namely, RNA transport, spliceosome, adherens junction, tight junction, ribosome biogenesis in eukaryotes, thyroid hormone signaling pathway, proteoglycans in cancer, focal adhesion, mRNA surveillance pathway, and Hippo signaling pathway. Furthermore, PDEPs were analyzed for GO, KEGG, and domain enrichment based on the difference expression multiplied PDEPs [Q1 (<0.667), Q2 (0.667–0.769), Q3 (1.3–1.5), Q4 (>1.5)] (Supplementary Figure 7).

Afterward, we used STRING and Cytoscape to visualize the interaction between PDEPs (Figure 5C) and used the MCODE plug-in in Cytoscape software to further analyze the PPI network and obtained 22 key and core proteins that were tighter in the network connection group (Figure 5D). We displayed the first four most compact MOCDEs (Figure 5D): MCODE 1 (MCODE score = 28.714) consisted of 29 nodes and 402 edges, MCODE 2 (MCODE score = 19.714) consisted of 50 nodes and 483 edges, MCODE 3 (MCODE score = 11.053) was composed of 20 nodes and 105 edges, and MCODE 4 (MCODE score = 10.727) was composed of 12 nodes and 59 edges. In addition, we chose the top 20 hub proteins in the PPI network based on the betweenness of the phosphorylated proteome (Supplementary Table 2), which usually plays a paramount role in network stability due to its high degree of connection/interaction.

### Motif Analysis of the Phosphosites

Protein phosphorylation modification is regulated by protein kinases, and different protein kinases prefer specific substrates with conserved motifs. We applied a large number of phosphorylation sites identified in this study for bioinformatics analysis, and we used the motif-x program to perform sequence analysis of the six amino acids upstream and six amino acids downstream of the identified phosphate sites of serine and threonine residues.

**FIGURE 6:**
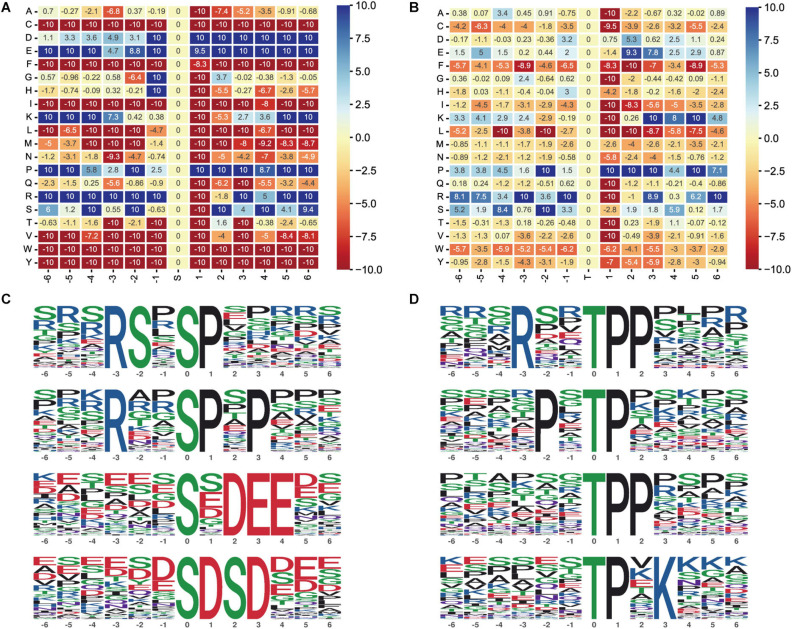
Motif analysis of the phosphosites. **(A)** Motif enrichment heatmap of phosphoserine. Blue indicates that this amino acid is considerably enriched near the modification site, and red indicates that this amino acid is fundamentally reduced near the modification site. **(B)** Motif enrichment heatmap of phosphothreonine. **(C)** Phosphoserine motif prediction ranking top four. **(D)** Phosphothreonine motif prediction ranking top four.

Moreover, we performed motif enrichment of phosphoserine (Figure 6A) and phosphothreonine (Figure 6B) in the form of a heatmap. The numbers on the abscissa represent the upstream and downstream phosphorylation modification sites. We identified 89 conserved motifs based on 1,068 phosphoserine (pS) phosphate sites and 15 conserved motifs and phosphoserine based on 104 phosphothreonine (pT) phosphate sites (Supplementary Table 1). We chose the top four hub motifs based on motif score, respectively (Figures 6C,D). The amino acids aspartate (D), glutamate (E), glycine (G), lysine (K), proline (P), and arginine (R) had a tendency to be present in the proximity of serine phosphosites. The amino acids glutamic acid (E), lysine (K), proline (P), arginine (R), and serine (S) had a tendency to be present in the proximity of threonine phosphosites.

### Kinase Prediction Network

**FIGURE 7:**
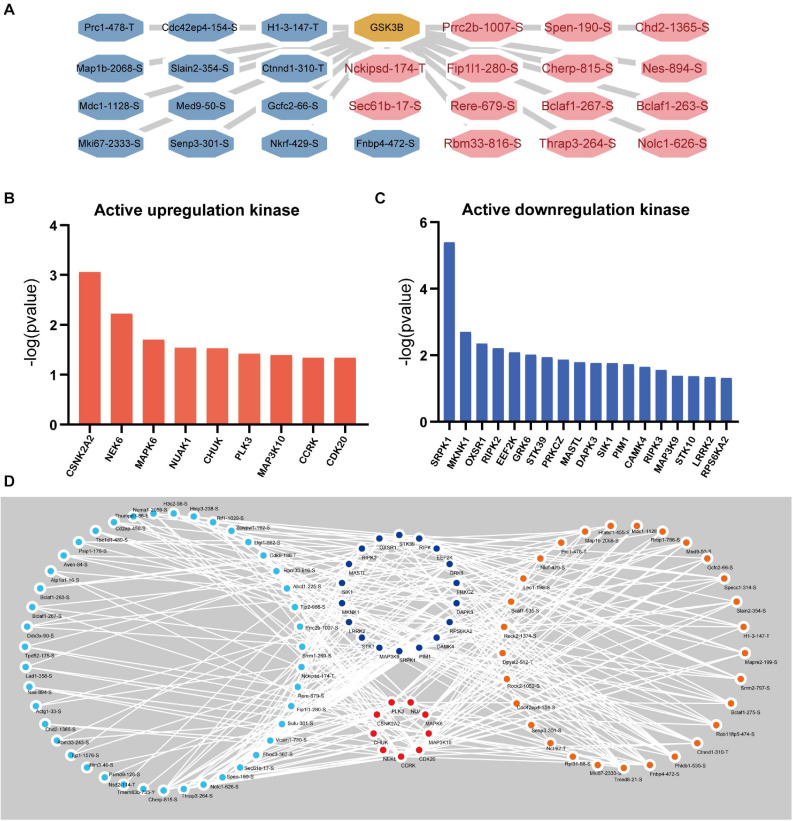
Kinase Prediction Network in podocytes. **(A)** GSK3β regulatory network. The GSK3β regulatory network was obtained by GPS5.0 (http://gps.biocuckoo.cn/) and Cytoscape (more than 1.8 times the phosphorylation site). **(B)** Active upregulation kinase. The kinase activity was upregulated, as calculated by the iKAP algorithm. **(C)** Active downregulation kinase. **(D)** Kinase regulatory network. Blue represents upregulated kinases, red represents downregulated kinases, orange represents increased phosphorylation sites (over 1.8 times), and sky blue represents decreased phosphorylation sites (under 0.56 times).

Apparently, the associated upstream kinase is unknown for the majority of the GSK-3β-regulated phosphosites. We therefore predicted the upstream kinase for each of these sites using the group-based prediction system (GPS) and iEKPD2.0. In accordance with the *p*-value of the identified protein ratio being under 0.05, 156,515 regulatory relationships between 299 protein kinases and 3,460 significantly changed phosphorylation modification sites on 1,574 proteins were identified (Supplementary Table 2). According to the prediction results, 1,299 regulatory sites of GSK-3β were obtained (Supplementary Table 3). In line with the changes in the expression level of the regulatory sites, the sites with a change of more than 1.8 times are displayed as shown in Figure 7A. The iKAP algorithm was used to obtain nine kinases with high levels (Figure 7B) and 18 kinases with low levels (Figure 7C). The activities of NIMA-related kinase 6 (NEK6) and casein kinase 2 alpha 2 (CSNK2A2) are apparently increased. The activity of SRSF Protein Kinase 1 (SRPK1) and MAPK Interacting Serine/Threonine Kinase 1 (MKNK1) are obviously decreased. Next, Cytoscape was used to demonstrate the activity of kinase regulatory networks (Figure 7D). Protein phosphorylation sites with a GSK3B-KD/control ratio of 1.8 times or more are displayed in the network.

## Discussion

Diabetic kidney disease (DKD) is a major cause of morbidity and mortality in diabetes and is the most common cause of end-stage renal disease (Warren et al., 2019). GSK-3β is a central regulator of proliferation, aging, apoptosis, and other physiological activities, and it also plays a key role in diabetic nephropathy. A recent study has established that a high expression of GSK-3β and its phosphorylated form are linked to the progression of albuminuria and diabetic kidney injury (Liang et al., 2020). In addition, the findings of Li et al. (2016) suggest that the β isoform of GSK3 mediates autonomous podocyte injury in glomerulopathy by integrating multiple podocytopathic signaling pathways. Moreover, GSK-3β has a novel functional role in polycystic kidney disease (PKD) pathophysiology, and its inhibition may be therapeutically useful to slow down cyst expansion and progression of PKD (Tao et al., 2015). There has been no quantitative analysis of the full range of GSK-3β-regulated functions. Yan Lu et al., confirmed the substrate of USP14 by proteome-wide quantitative analysis of the USP14-regulated proteome, ubiquitinome, and interaction (Sharma et al., 2020). Consequently, it is necessary to analyze the regulatory network involved in GSK-3β by proteomic and phosphoproteomic analyses.

Our proteome analysis identified 115 proteins with lower levels and 34 proteins with higher levels in response to GSK-3β, which revealed the first landscape of GSK-3β participating in cellular pathways and networks. Our results not only identified the well-recognized functions of GSK-3β, such as axon guidance and apoptosis, but also revealed its new potential roles in the spliceosome and Hippo signaling pathways, suggesting a broad regulatory role of GSK-3β in various cellular functions. Consistently, our ubiquitinome analysis showed that GSK-3β-associated phosphorylation plays a critical role in a variety of cellular processes, especially in cell growth, cell migration, and axon extension involved in axon guidance.

Notably, this research demonstrated that the phosphorylation levels of Trhde, Spp1, and Mical3 were reduced and the total protein expression levels were increased. TRH-degrading ectoenzyme (Trhde) was discovered for the first time to interact with GSK-3β. Some studies have shown that under high blood sugar conditions, diabetic mice and podocyte osteopontin (OPN/spp1) are significantly increased (Zou et al., 2017). In addition, OPN can prevent apoptosis and stimulate the proliferation of islets and insulin-producing cells (Lyssenko et al., 2011). Our research highlights that GSK-3β, as a kinase, can directly or indirectly phosphorylate OPN. Therefore, we attribute that GSK-3β may mediate the apoptosis and proliferation of podocytes by OPN.

The identified GSK-3β-dependent phosphoproteins span a wide array of functions, including spliceosome proteins, RNA transport factors, and microtubule-associated proteins. The spliceosome is a multicomponent complex that plays a role in the assembly of newly synthesized pre-mRNA (precursor messenger RNA), thereby interfering with normal protein synthesis and causing disease (van Alphen et al., 2009). In our study, differentially phosphorylated proteins were enriched in the spliceosome pathway. It has been reported that RBM17 (up: 1.366-fold at Ser155) is essential for survival and cell maintenance and interacts with the spliceosomal factors U2SURP (down: 0.575-fold at Ser974, 0.652-fold at Ser969, 0.705-fold at Try919, 0.75-fold at Ser49) and CHERP (up: 2.094-fold at Ser815) and that they reciprocally regulate each other’s stability (De Maio et al., 2018). In addition, inhibiting the expression of U2surp (down: 0.575-fold at Ser974) inhibits cell colony formation and significantly slows cell growth in breast cancer cells (An et al., 2020). Acin1 (up: 1.419-fold at Ser561, 1.331-fold at Ser479, 1.318-fold at Ser863, 1.309-fold at Ser825; down: 0.737-fold at Ser667, 0.71-fold at Ser583, 0.7-fold at Ser491) is associated with apoptosis (Sahara et al., 1999). These observations suggest that GSK-3β-dependent phosphorylated proteins may be involved in the formation of shears and thus affect protein synthesis.

**FIGURE 8:**
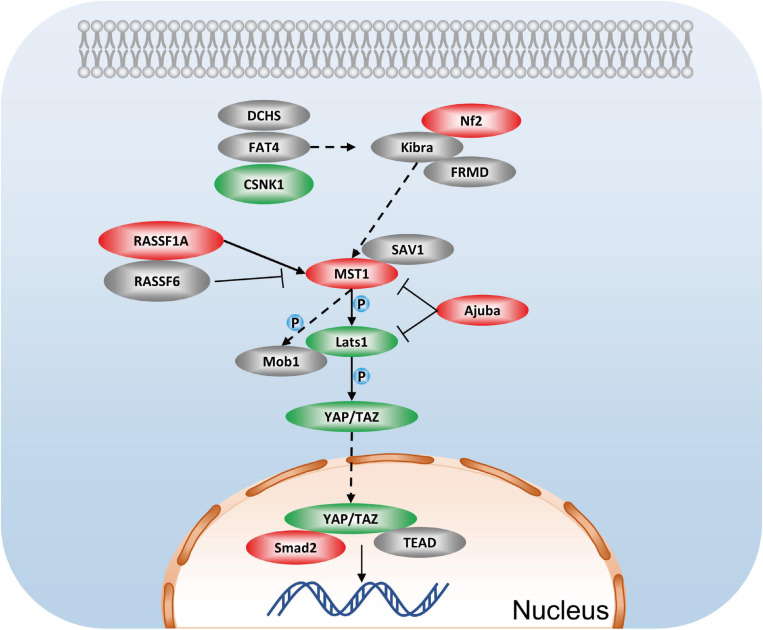
Hippo signaling pathway in podocytes after GSK3β knockdown. Red represents the phosphorylation proteins with higher levels in the lentivirus-mediated GSK-3β knockdown group than in the control group, green represents the phosphorylation proteins with lower levels, and gray represents no difference detected proteins.

One of the most striking findings in the phosphoproteomic data set is the identification of multiple proteins involved in the Hippo signaling pathway, including Smad2 (up: 1.627-fold at Thr8), Stk3 (Mst1), Nf2 (up: 1.482-fold at Ser518), lats1 (down: 0.727-fold at Ser204), Ajuba (up: 1.33-fold at Ser272, 1.467-fold at Ser239, 1.366-fold at Ser147), CSNK1E, RASSF1, Scrib, and others (Figure 8). Hippo signaling has proven that inhibiting Hippo signaling could be a potential therapy for renal fibrosis (Lei et al., 2019). Studies of YAP suggest that YAP promotes cell survival by inhibiting proapoptotic dendrin signaling (Campbell et al., 2013). Additionally, YAP activation in renal proximal tubule cells drives diabetic renal interstitial fibrogenesis (Chen et al., 2020). Studies on NF2 have provided evidence that it can regulate the activity of Hippo, thereby modulating cell proliferation and apoptosis (Hamaratoglu et al., 2006). Recently, research contends that the lack of renal tubules Mst1/Mst2 can lead to CKD through the YAP and non-YAP pathways, and the activation of renal tubules YAP contributes to renal fibrosis (Xu et al., 2020). Combining previous research and our unbiased phosphoproteome analysis, we inferred that GSK-3β may mediate apoptosis and cell proliferation by the Hippo signaling pathway.

Our analysis of phosphorylation *in vivo* cannot distinguish direct phosphorylation and indirect phosphorylation, so we adopted the iKAP (18) algorithm to predict kinase activity based on the expression of phosphorylation sites. Our kinase network predictions manifest the phosphorylation effect of GSK-3β on Map1b, Nes, Mki67, etc. Jeffrey W. Pollard et al., argued that LiCl, as a selective inhibitor of GSK-3β, can inhibit the increase in mki67 caused by 17B-estradiol (Polotsky et al., 2009). Several studies have illustrated that depletion of GSK-3β diminishes the phosphorylation of MAP1B (Laks et al., 2018). Prior research suggests that the expression of NES is altered by some compounds partly by regulating GSK-3β signaling (Liang et al., 2019). Additionally, Mdc1 was detected in our study to be phosphorylated by GSK-3β, but there is no other relevant research yet. Furthermore, we performed kinase activity predictions and found that the activity of 27 kinases changed after GSK-3β was knocked down. There is no relevant research at present, and this prediction provides new ideas for GSK-3β research.

While our results are encouraging, we are also aware of the limitations of our study. The technologies employed in this study have their own limitations. Current MS-based proteome analysis still cannot achieve complete coverage of the highly dynamic cellular proteome. Therefore, our quantitative proteome analysis may miss low-abundance proteins. In addition, our research is limited to intracellular labeling and has not been further verified in a clinical setting. Further experiments are needed to consolidate the conclusions drawn from proteomics research data. Future studies will test the downstream regulatory network of GSK-3β either individually or in combination.

In general, this work provides unique prospects and resources for future research on GSK-3β. The functions of these proteins were determined according to our analysis. These differentially expressed proteins may be employed as therapeutic targets in diabetic nephropathy.

## Data Availability Statement

The data presented in the study are deposited in the ProteomeXchange Consortium via the PRIDE partner repository, accession number (PXD024543).

## Author Contributions

MH performed the experimental design, implemented the experiments, performed cell culturing, lentiviral transfection and stable transformation construction, and western blot experiments, analyzed the data, participated in the interpretation of the results, and wrote the manuscript. SZ and JF designed the experiments and participated in the implementation of the experiments, interpretation of the results, and preparation of the manuscript. HW was involved in cell culturing. SZ initiated the study and read and approved the manuscript. All authors read and approved the final manuscript.

## Conflict of Interest

The authors declare that the research was conducted in the absence of any commercial or financial relationships that could be construed as a potential conflict of interest.

## Footnotes

1 http://www.ebi.ac.uk/GOA/

2 http://string-db.org

3 http://apps.cytoscape.org/apps/mcode

4 http://gps.biocuckoo.cn/
