# Supplementary material for: Proteome and Phosphoproteome Analyses Reveal the Kinase Regulatory Network Involved in Glycogen Synthesis Kinase 3β
Source: Front Genet. 2021 Apr 7;12:657140. doi: 10.3389/fgene.2021.657140 (PMC8059835; doi:10.3389/fgene.2021.657140)
Supplement: Supplementary file 1 [file Data_Sheet_1.PDF]

Supplementary Figures

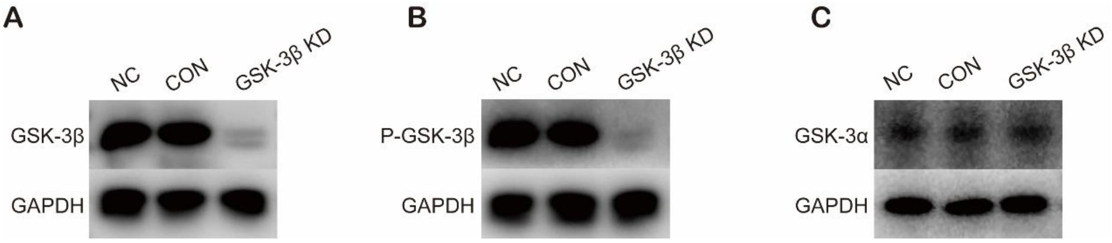

Figure S1. The protein expression levels of GSK-3β, p-GSK-3β and GSK-3α were determined by Western blot.

(A) Representative protein levels of GSK-3β in podocytes administrated with lentivirus or not as indicated. (B) Quantification of p-GSK3β protein levels relative to GAPDH protein levels in different groups. (C) Representative Western blots showing the expression of the GSK-3α in the knockdown of GSK-3β podocytes.

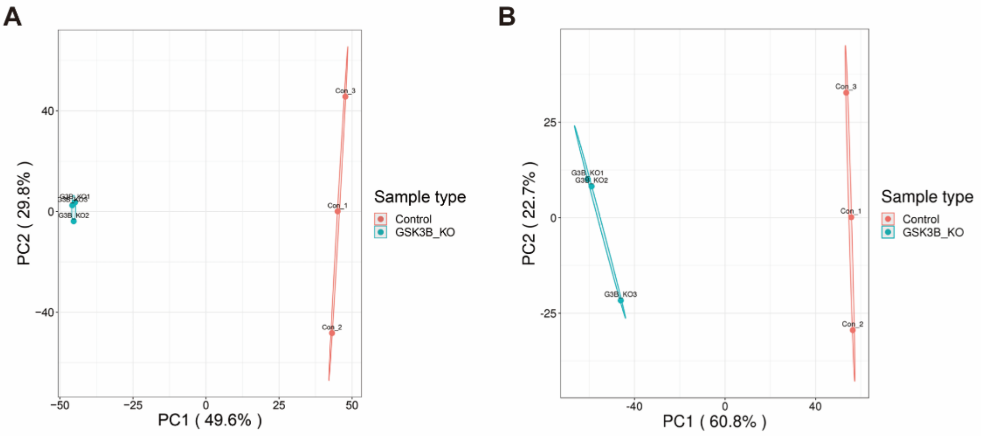

Figure S2. (A) PCA analysis of quantitative proteome. (B) PCA analysis of phosphorylated proteome.

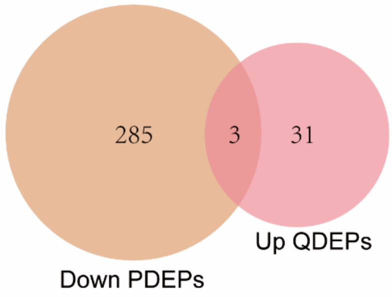

Figure S3. Venn diagram of downregulated PDEPs and upregulated QDEPs.

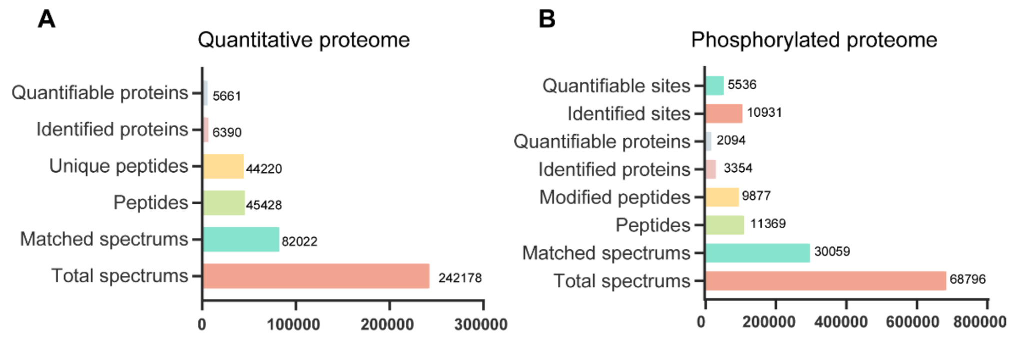

Figure S4. (A) MS/MS spectrum database search analysis of quantitative proteome. (B) MS/MS spectrum database search analysis of phosphorylated proteome

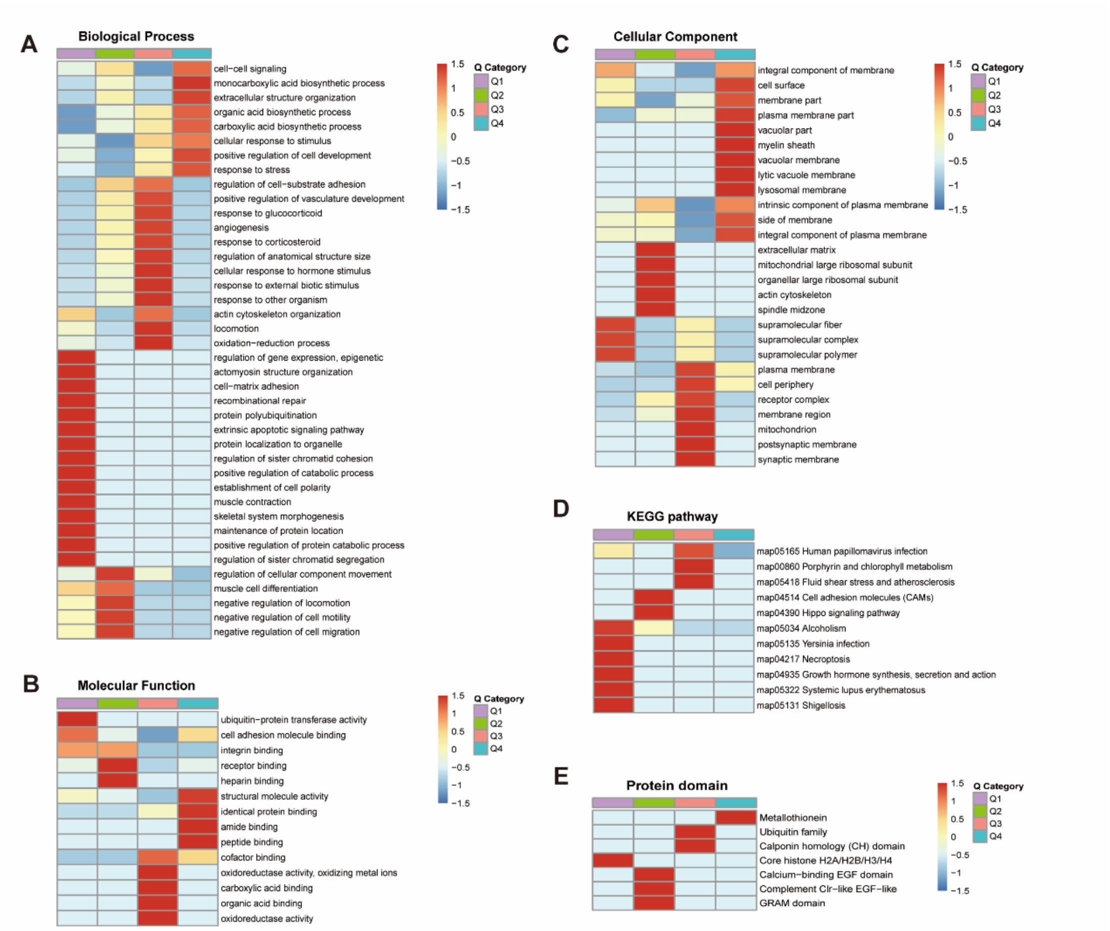

Figure S5. GO, KEGG, and domain enrichment based on the QDEPs (Q1(<0.667), Q2(0.667-0.769), Q3(1.3-1.5), Q4(>1.5))

(A) Functional enrichment clustering analyses of QDEPs in four quantiles (Q1-Q4) based on biological process. (B) Functional enrichment clustering analyses of QDEPs in four quantiles (Q1-Q4) based on molecular function. (C) Functional enrichment clustering analyses of QDEPs based on cellular component. (D) Kyoto Encyclopedia of Genes and Genomes (KEGG) enrichment clustering analyses of QDEPs in four quantiles (Q1-Q4). (E) Functional enrichment clustering analyses of QDEPs in four quantiles (Q1-Q4) based on protein domain.

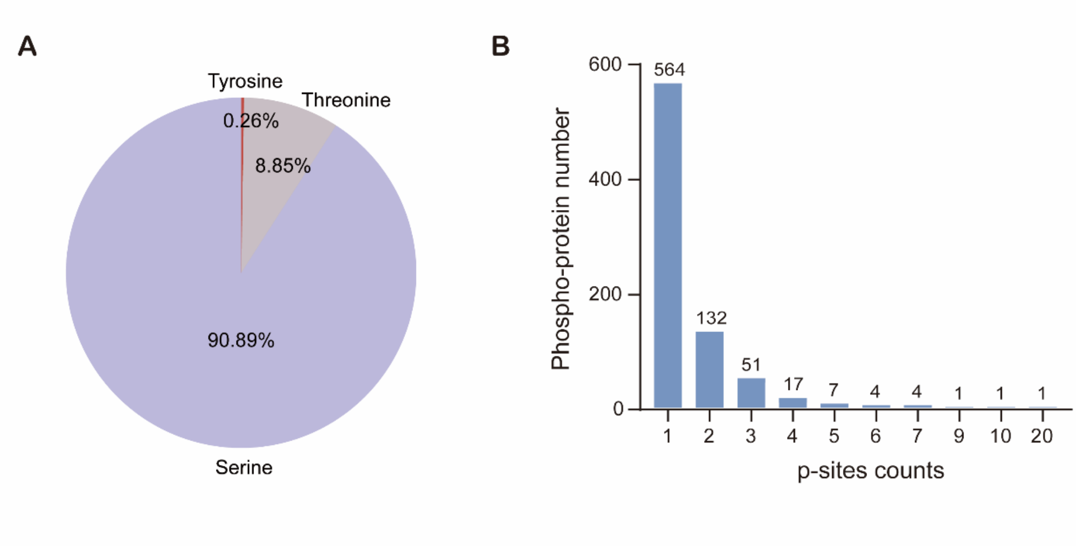

Figure S6. The quantitation and identification of the phosphoproteome of podocytes in response to GSK-3β knockdown.

(A) Distribution of the Serine, Threonine and Tyrosine phosphorylation events by global phosphoproteomics. (B) Overview of phosphorylation sites per protein. Distribution of phosphoproteins based on number of phosphorylation sites per protein.

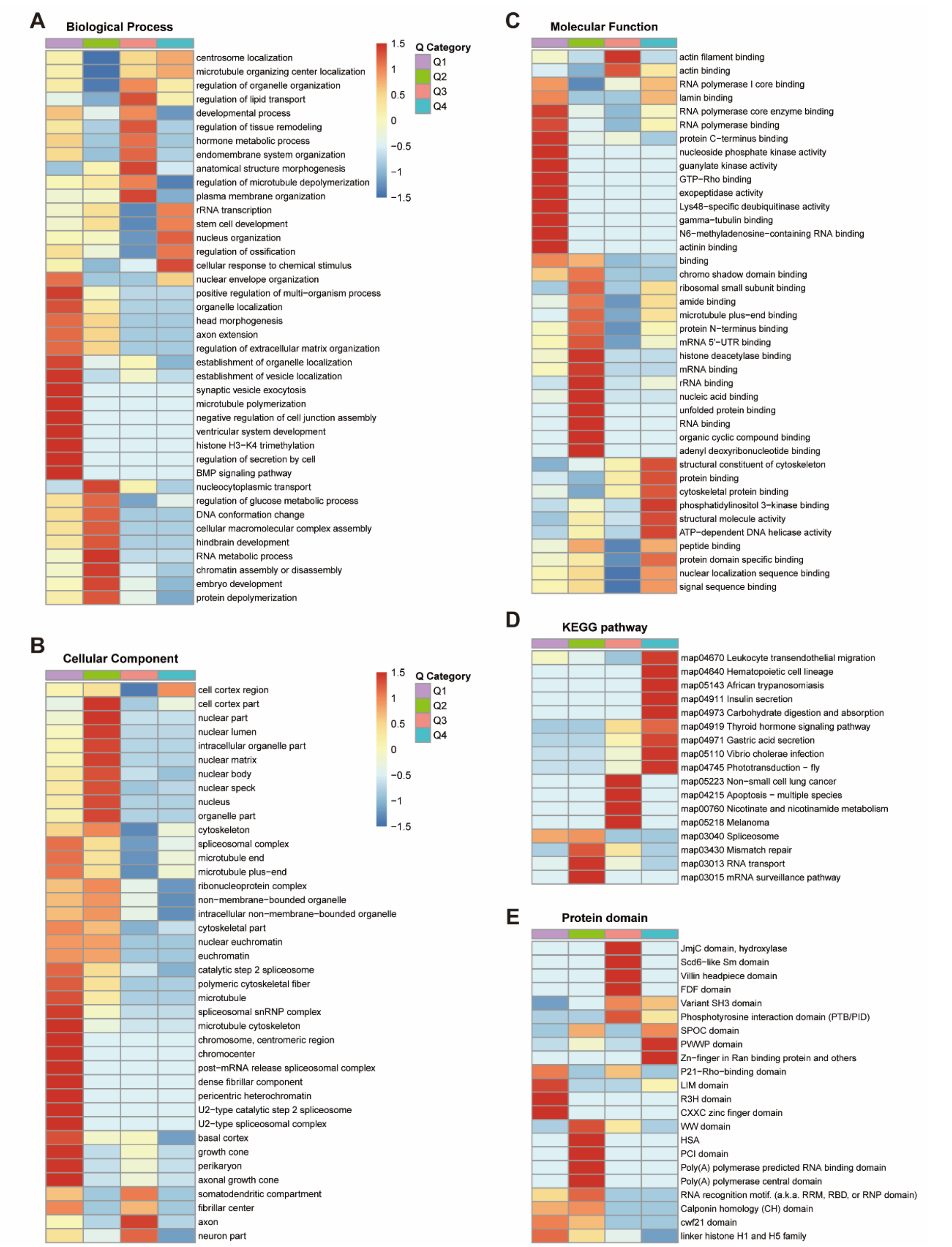

Figure S7. GO, KEGG, and domain enrichment based on the PDEPs (Q1(<0.667), Q2(0.667-0.769), Q3(1.3-1.5), Q4(>1.5))

(A) Functional enrichment clustering analyses of PDEPs in four quantiles (Q1-Q4) based on biological process. (B) Functional enrichment clustering analyses of PDEPs in four quantiles (Q1-Q4) based on molecular function. (C) Functional enrichment clustering analyses of PDEPs based on cellular component. (D) Kyoto Encyclopedia of Genes and Genomes (KEGG) enrichment clustering analyses of PDEPs in four quantiles (Q1-Q4). (E) Functional enrichment clustering analyses of PDEPs based on protein domain.

Supplementary Tables

Table S1. Top 20 hub proteins in the PPI network based on the Degree of quantitative proteome

Gene name	Degree	Betweenness	Closeness	Centroid	Bridging
Hmox1	10	891.654	0.004202	-59	20.21564
Isg15	8	848.6413	0.004587	-59	44.33201
Ndufab1	8	822.7413	0.004329	-59	29.65597
Ube2e1	8	1062.937	0.004926	-59	44.76869
Mrpl2	8	459.4984	0.004049	-59	33.59842
Spp1	8	373.4683	0.003906	-59	20.81431
Cav1	7	1408.059	0.004608	-59	78.08089
Rnf14	7	782.519	0.004739	-59	45.62793
Gsk3b	7	576.654	0.004149	-59	48.42441
Rps28	7	184.1016	0.003906	-59	16.78738
Oasl1	6	141.7048	0.003984	-59	19.26084
Smad2	6	147.1095	0.004098	-59	18.2421
Mrpl4	5	93.33333	0.003745	-59	22.14689
Sil1	5	232	0.003344	-59	38.09971
Rnf41	5	1356.059	0.004975	-59	213.9135
Bst2	4	405.9413	0.004149	-59	115.9832
Ifit1	4	5.833333	0.003759	-59	1.666667
Eif5a	4	0	0.003322	-59	0
Fbxl4	4	0	0.004425	-59	0
Fbxo11	4	0	0.004425	-59	0

Degree, Betweenness, centroid, closeness and bridging are algorithms provided by plugin Centiscape2.2 in software Cytoscape.

Table S2. Top 20 hub proteins in the PPI network based on the Degree of phosphorylated proteome

gene name	Degree	Betweenness	Closeness	Centroid	Bridging
Trp53	144	67002.95	6.49E-04	130	46.68626
Ncbp1	92	11758.55	5.45E-04	-243	32.63136
Hsp90aa1	81	20717.05	5.98E-04	-130	56.32297
Egfr	77	25201.94	5.80E-04	-183	61.50255
Ranbp2	73	19431.16	5.79E-04	-187	67.71898
Hspa8	72	16804.31	5.86E-04	-166	90.77142
Srsf1	72	10480.74	5.61E-04	-240	49.4104
Ddx5	71	7048.399	5.58E-04	-251	49.93468
Polr2a	69	18011.56	5.69E-04	-196	76.31636
Hsp90ab1	69	14858.7	5.90E-04	-153	56.51497
Ccnd1	67	11803.15	5.68E-04	-219	52.00644
Hnrnpa1	64	5720.527	5.62E-04	-239	46.00335
Rnps1	63	3692.591	5.15E-04	-320	25.32776
Cdc20	61	6674.663	5.34E-04	-330	42.15321
Nop56	59	5811.573	5.29E-04	-297	42.01919
Sf3b1	59	8104.741	5.32E-04	-281	53.56425
Ncl	59	7471.938	5.49E-04	-282	54.02091
Hnrnpa2b1	59	3426.673	5.34E-04	-284	29.32681
Crebbp	59	10701.94	5.61E-04	-240	43.55058
Ezh2	58	7334.118	5.46E-04	-292	40.28934

Degree, Betweenness, centroid, closeness and bridging are algorithms provided by plugin Centiscape2.2 in software Cytoscape.
